# Those who can’t sort, steal: caste, occupational mobility, and rent-seeking in rural India

**DOI:** 10.1017/dem.2020.21

**Published:** 2021-03-01

**Authors:** Nicholas Lawson, Dean Spears

**Affiliations:** 1 Département des sciences économiques, Université du Québec à Montréal, Case Postale 8888, Succursale Centre-Ville, Montréal, Québec, H3C 3P8, Canada; 2 Department of Economics, University of Texas at Austin, 2225 Speedway, BRB 1.116, C3100, Austin, 78712, Texas, USA; 3 Indian Statistical Institute - Delhi, Delhi, India; 4 IZA, Bonn, Germany; 5 IFFS, Stockholm, Sweden

**Keywords:** Caste, India, occupational mobility, rent-seeking

## Abstract

Three important features of Indian labor markets enduringly coexist: rent-seeking, occupational immobility, and caste. These facts are puzzling, given theories that predict static, equilibrium social inequality without conflict. Our model explains these facts as an equilibrium outcome. Some people switch caste-associated occupations for an easier source of rents, rather than for productivity. This undermines trust between castes and shuts down occupational mobility, which further encourages rent-seeking due to an inability of workers to sort into occupations. We motivate our contribution with novel stylized facts exploiting a unique survey question on casteism in India, which we show is associated with rent-seeking.

## 1. Introduction

A large and important literature in development economics considers the consequences for the development of productive versus extractive/rent-seeking institutions.^1^ Another literature focusses on the economic consequences of inequality: do economically unequal countries have worse growth?^2^ We analyze the important case of caste in India, a cultural context in which both of these questions are significant [Hoff and Stiglitz (2016)]—in which extractive, immiserating institutions are endogenously co-determined with social and horizontal inequality.^3^

In particular, this paper is concerned with three important features of modern India: rent-seeking, occupational immobility, and caste. Rent-seeking is a significant problem in India, as argued by The Economist (March 15, 2014). Meanwhile, a substantial empirical literature demonstrates that occupational mobility is very low in India: Hnatkovska et al. (2013) find that only 41% of their sample in 2004–05 worked in a different 3-digit occupation from their household head.^4^ And finally, caste remains one of the defining features of India’s social structure: Indians are divided both vertically (into broad classes known as *varna*) and horizontally (into sub-caste groups known as *jati*) on caste lines, into social groups that are hereditary and often endogamous.^5^ Akerlof (1976) provides an early analysis of the economic inefficiency of caste as a social equilibrium that shuns and punishes those who break caste customs.

We argue that these three features are inter-related in an important and previously unrecognized way. In particular, we motivate our analysis with a series of novel stylized facts from the 2012 round of the India Human Development Survey (IHDS), in which we are able to use a unique question on household practices of untouchability. Using the village average of this variable as an approximate measure of *casteism*, or the local strength of the social forces of caste, we find that villages where caste is more important feature less occupational mobility and more rent-seeking (in the form of theft and threats, and to a lesser extent village conflict). We further find evidence that casteism is associated with lower returns to education, lower consumption, and lower geographical mobility.

We further argue that these results are collectively puzzling, in light of prominent theories in the literature. A correlation between casteism and occupational mobility is not surprising, because *jati* are traditionally associated with an occupation, and this particular result is consistent with previous empirical analysis. However, the association between casteism and rent-seeking is not at all obvious: places where caste is more important might be thought to be more socially conservative places in which one might expect individuals to “know their place”. The model in Akerlof (1976) cannot explain this outcome, as it is a model of self-fulfilling expectations in which nobody is off the equilibrium path so that punishment and/or violence never happens; indeed, Akerlof’s model is entirely consistent with the aforementioned social conservatism story.^6^ Meanwhile, many alternative models of social division would similarly suggest that theft and other forms of rent-seeking would be less common in areas suffering from more discrimination: a taste-based model of discrimination as in Becker (1957) would imply that poor people are less able to indulge in discrimination—and caste tends to be more important in poorer parts of India.

Therefore, in the main part of our paper, we provide a new theoretical model which can explain our empirical findings, and which we can subsequently use to consider extensions and policy options. Specifically, we present a model in which individuals are of multiple types, which face differing incentives to engage in predatory rent-seeking, and in which there is a utility cost of rent-seeking which is larger when an individual steals within the occupation associated with their own caste, where we will use the word “caste” to refer to horizontally-differentiated *jati*. We motivate this assumption by the idea that there is an added risk from rent-seeking within one’s own caste: a *jati* provides a network of mutual insurance to its members, as demonstrated by Munshi and Rosenzweig (2016), and it seems plausible that rent-seeking within one’s *jati* carries a risk of being cut off from the insurance network, as well as from other valuable social benefits.

An implication of this assumption is that, if occupations are traditionally associated with castes, some individuals who want to switch occupations do it not for productive reasons, but rather to find an easier or less costly context to engage in rent-seeking. In our model, this generates a lack of trust between castes and makes people unwilling to interact with people from other castes.^7^ Thus, the existence of caste, combined with a sufficiently low utility cost of rent-seeking, shuts down occupational mobility. Finally, in the absence of occupational mobility, some individuals will be stuck in an occupation—the one associated with their caste—that does not suit their abilities, and we show that in a significant region of parameter space, this leads to even more rent-seeking than in a setting without caste identities.

Therefore, our model predicts that the existence of caste can generate both occupational immobility (due to fear of rent-seeking from other castes) and increased rent-seeking (due to the low productivity of some workers who are unable to sort into a suitable occupation). These results are consistent with our stylized facts from the IHDS. To be clear, our empirical analysis is not causal in nature, as we cannot claim that the local strength of caste is exogenous to other unobserved factors which may influence outcomes such as occupational mobility or rent-seeking. Rather, we interpret our stylized facts as correlations (conditional on a variety of controls) that are endogenous equilibrium outcomes of the real data-generating process, just as our theoretical results are endogenous equilibrium outcomes of our model.

In a final section of the paper, we present a series of extensions of our model, including an analysis of possible policy implications of our analysis. We begin by adding a general utility cost from interacting with other castes—whether productively or for rent-seeking—and we show that, surprisingly, this dislike of cross-caste interaction can increase the probability of attaining the efficient equilibrium. This conclusion stands in contrast to the famous “contact hypothesis” of the social sciences [Allport (1954)], but so does the endurance of casteism in Indian villages and cities dense with caste heterogeneity. Essentially, in this setting, a worker would only switch occupations/castes in order to receive a large income gain; in other words, this new utility cost deters cross-caste rent-seekers, leaving only those individuals whose productivity is much higher in the other occupation wanting to switch, encouraging cooperation as the risk of rent-seeking is diminished. This extension can explain why an inefficient high-rent-seeking equilibrium is particularly likely in a highly geographically dense and integrated country such as India: the utility cost of migrating to interact with other castes is very small, and indeed we show that our empirical results are even stronger in districts where population density is greater.

A second extension considers the role of education and shows that a lowered return to education when caste is important is another natural implication of our model, because casteism encourages occupational immobility which limits the return to specialized education, and because casteism can generate predatory rent-seeking which directly reduces the return to investment in productive capabilities. Finally, we extend our model to consider the role of redistribution: we show that if rent-seeking is sufficiently costly in utility terms and if the government is able to sufficiently tax income from rent-seeking as well as productive work, redistribution would be predicted to reduce rent-seeking, thus reducing the segregation of castes and encouraging occupational mobility and efficiency.

Our model contributes to several theoretical literatures. Most importantly, our paper is complementary to the work of Munshi and Rosenzweig (2016) on misallocation of labor and caste networks of mutual insurance; here, the misallocation is across occupations rather than space, and the mechanism is different: rent-seeking does not appear in Munshi and Rosenzweig (2016). Another literature considers conflict within and between groups, and our results effectively invert the logic found in Münster and Staal (2011), who find that groups have an incentive to fight with other groups to exhaust any desire to fight within-group; in our context, we find that an inability to “fight” with outsiders can intensify internal fighting, in the form of rent-seeking.

Our model also contributes to literature on the factors that sustain the social force of caste; in particular, Bidner and Eswaran (2015) and Choy (2018) explain the endurance of caste through its beneficial effects on certain outcomes. Bidner and Eswaran (2015) argue that endogamy can be efficient when women complement their husband’s occupation-specific skills, whereas Choy (2018) presents a model in which caste segregation increases trust and cooperation. Our paper is complementary, in that we emphasize the horizontal differentiation of castes rather than the vertical dimension, and we present a negative reason for the persistence of caste segregation: we show that caste can generate an inefficient and yet stable equilibrium characterized by immobility and rent-seeking, which is consistent with the empirical correlations.

Our stylized facts are also related to a variety of papers in an empirical literature on the effects of caste on cross-caste interactions. For example, Anderson (2011) provides evidence of breakdowns in trade between caste groups which lead to higher incomes for low-caste households when a lower caste dominates their village,^8^ while Jacoby and Mansuri (2015) find that children in Pakistan—especially those from lower castes, and girls in particular—are unlikely to attend schools located in settlements dominated by other castes.

The rest of the paper proceeds as follows. Section 2 presents and discusses a series of new stylized facts about caste, with a particular focus on occupational immobility and rent-seeking. Section 3 then presents the main model and derives results, while section 4 considers a number of extensions of the model and possible policy options. Section 5 concludes the paper.

## 2. Motivation: stylized facts

Following from Akerlof (1976), a variety of empirical papers touch on caste and a dimension of economic or social interactions. Unlike these prior papers that investigate households’ or workers’ own caste status or identity (perhaps in combination or interaction with other persons’ caste identities), we study a novel measure of *casteism*, the attitude or posture that supports caste-based discrimination and its enforcement. In other words, our focus is not on differences in outcomes between members of different castes; it is on differences for everybody between places where caste is more or less important. This will be a key feature of our model.

**Figure 1.**
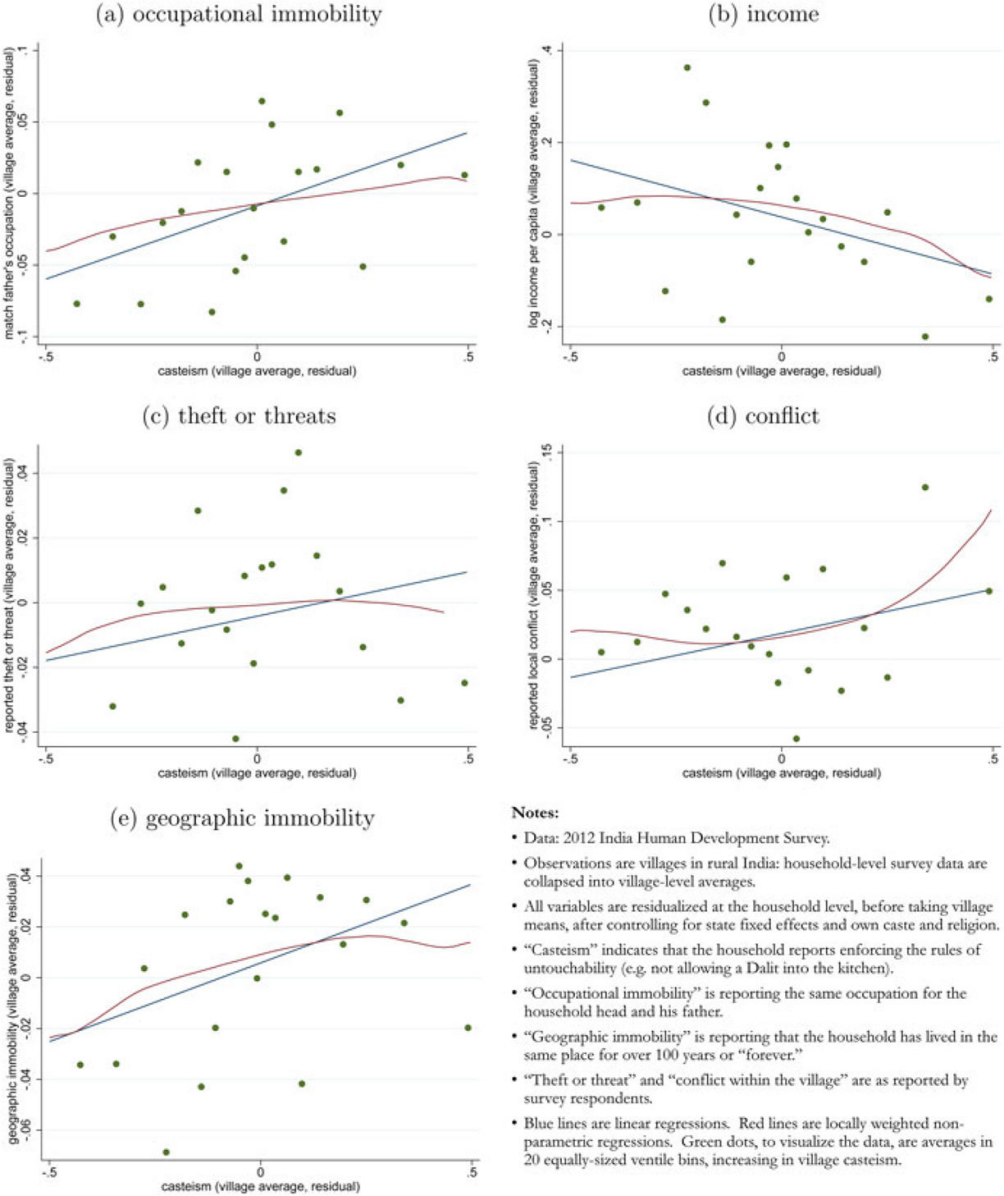
Stylized facts: casteism across rural Indian villages.

We add to this empirical literature with results in Figure 1. In each panel of this figure, the horizontal axis plots a measure of village-level casteism: the mean number of households in a village that report enforcing the rules of untouchability in their interactions with others.^9^ Given the salience of such customs of untouchability, we take this variable as an indicator of the strength of the social forces of caste in the village: where more people follow the rules of untouchability, caste is more important. This analysis is possible due to a unique set of survey questions that asked about untouchability in the 2012 round of the Indian Human Development Survey (IHDS). We use only rural households from the IHDS [Desai and Vanneman (2018)]. Each observation is a single collapsed village; we analyze these data at the village level because we are interested in equilibrium outcomes in local labor markets. In Appendix A we confirm that none of these results is due to the caste, religious, occupational, or educational composition of villages.

### Stylized Fact 1.

Casteism occurs alongside occupational and geographic immobility.

This is visible in panels (a) and (e). Panel (a) shows that villages in which more households report enforcing untouchability are villages where household heads are more likely to work in the same occupation as their fathers. Panel (e) shows that villages with more reported casteism are villages where household heads are more likely to report that their household has lived in the same home “forever” (rather than having moved to their home). Urbanization and permanent migration more broadly (other than of women for marriage) are both known to be unusually uncommon in India, plausibly due to the social capital and ties of the caste system [Munshi and Rosenzweig (2016)].

### Stylized Fact 2.

Casteism occurs alongside poverty and lower-income.

This is visible in panel (b): household incomes are lower, on average, in villages where more people report enforcing the rules of untouchability. This is consistent with a classic understanding of the consequences of discrimination in economics: discrimination can be costly and unproductive because workers are not horizontally matched to the occupations where they would be most productive.

### Stylized Fact 3.

Casteism occurs alongside rent-seeking, conflict, and crime.

This is visible in panels (c) and (d), which show that intra-village conflict (as reported in a survey section on social relations within the village) and crime (as measured by household reports of “theft” or “threats” in a survey section on crime) are both more common in villages where more survey respondents report casteist social attitudes.^10^ Given that we will model rent-seeking as a predatory activity in our model, we think that these variables—particularly the measure of theft and threats—are accurate representations of the kind of rent-seeking that we want to capture.^11^

Casteism paradoxically combines stasis with conflict: economic arrangements endure alongside equally enduring contestation and appropriation. Thus, it is not merely true that casteism is associated with an occupational *difference* or even with low productivity and income; it is also associated with ongoing conflict and rent-seeking, in a way that cannot be accounted for by mere equilibrium horizontal differentiation.

The rest of this paper focuses on our main contribution: a theoretical account of caste and casteism that accounts for these stylized facts as equilibrium outcomes. For more detail on the variables and data used in Figure 1, and for an in-depth investigation of the robustness of these associations in a regression framework, please see Appendix A.

## 3. Model & results

We now present a simple discrete-type model of production, to investigate how the forces of caste can generate both occupational immobility and rent-seeking. Our model follows the basic concept of rent-seeking defined and formalized by Murphy et al. (1991), in which rent-seeking is predatory and involves stealing uniformly from productive individuals. In Murphy et al. (1991), individuals have a choice between productive work (as an entrepreneur or a worker) and rent-seeking in a single-sector economy, and rent-seekers steal uniformly from the profits of entrepreneurs within that same sector, whereas in our model we generalize this model to two sectors: individuals have a choice between occupations (or sectors of the economy), and then within an occupation, they must choose between productive work and rent-seeking as in Murphy et al. (1991).^12^ We focus on predatory rent-seeking with the goal of matching our model as closely as possible to the stylized facts in section 2, particularly the definition there of theft and threats as a form of rent-seeking.

The first subsection presents the setup of the model, while the second discusses parametric assumptions we make to simplify the algebraic analysis, and the third subsection presents the solution for the equilibrium. Finally, a fourth subsection discusses the effects of caste on occupational immobility and rent-seeking, to identify how our theoretical results connect to the stylized facts from section 2. Our model derives equilibrium allocations from individual choices in a setting where rent-seeking involves stealing uniformly from a given caste’s occupation, but Appendix D shows that the same basic results hold in a setting in which rent-seeking involves stealing from an individual team member within an occupation.

### 3.1 Model setup

Individuals in our model are divided into two castes, 1 and 2, which are horizontally differentiated only in the sense that each is associated with a particular occupation.^13^ We abstract from the hierarchical aspect of the caste system in order to focus on the horizontal division of society into separate sub-caste or *jati* groups. The hierarchy of the caste system is also of considerable importance along numerous dimensions and may be relevant for rent-seeking as well, but our goal is to demonstrate that the pervasive feature of rent-seeking can be parsimoniously explained by the division of Indian society into thousands of *jati* sub-groups, many of which are ambiguously ranked relative to each other.^14^ While our stylized facts use a measure of casteism that is related to vertical discrimination, it is the only direct measure of casteism that we are aware of in India, and so it is our best possible measure of the strength of the caste system as a force in local society.^15^

While we will refer to occupations, the intuition is more general: the model is isomorphic to one in which individuals who “switch occupations” simply choose to have some economic interaction with individuals from another caste (consistent with the stylized fact of geographic immobility), and thus have the possibility of stealing from a different caste community. Our mechanism thus emphasizes that it is the possibility of interacting with members from different groups which may lead to rent-seeking, and we focus on predatory rent-seeking as in Murphy et al. (1991). In particular, we model rent-seeking as closely as possible to the way it is represented in the stylized facts section: theft at the community level, though of course, we cannot observe whether such rent-seeking takes place between or across castes in the data. Obviously, a great deal of rent-seeking in India may take place in quite different contexts from what we model, and we abstract from these other sources of rent-seeking.^16^ We limit the number of castes to 2 to simplify the algebra, but the underlying mechanism would continue to apply in a more general model.

In our model, an individual’s type is described by three binary variables: caste c={1,2}; output in their own caste’s occupation $z_{c}=\{1,2\}$; and output in the other caste’s occupation $z_{f}=\{1,2\}$. We assume a uniform distribution so that each caste contains 50% of the overall population, and that within each caste one-quarter of the population is of each of 4 productivity types: $\left\{z_{c},z_{f}\}=\{\{1,1\},\{1,2\},\{2,1\},\{2,2\}\right\}$. Caste is observable, but an individual’s productivity type is their own private information.

Each individual must decide (i) which occupation to choose, and (ii) whether to work productively or engage in rent-seeking.^17^ We assume that individuals are raised with the basic knowledge of how to perform their own caste’s occupation, but an individual who chooses the occupation associated with the other caste must learn from a member of the latter caste in order to enter the occupation, and this “teacher” is assigned randomly and can provide the necessary skills costlessly. However, the teacher has the option of refusing to help the entrant, which they may choose to do if they expect the entrant to engage in rent-seeking.

Before describing the utility functions, we describe the timing of the game, and to simplify the analysis of subgame perfect equilibrium, we model the timing as follows:

- *Stage 1:* All individuals decide whether they are willing to cooperate with members of the other caste: conditional on being matched with an entrant from the other caste in stage 3, they decide whether they will accept to help the entrant.
- *Stage 2:* All individuals choose an occupation, and whether to work productively or engage in rent-seeking from the members of that occupation, and pay a utility cost for this choice. The choice of occupation is visible to everyone, but the choice of productive work or rent-seeking is private information of the individual.
- *Stage 3:* Each entrant from caste i into occupation j≠i is matched with a member of caste j, and the entrant successfully enters the occupation if the “teacher” chose cooperation (i.e., accepted to provide the necessary skills) in stage 1. Production and rent-seeking take place, and individuals receive utility from consuming their resulting incomes. Entrants who were refused entry into the occupation associated with the other caste are in neither occupation, produce/steal nothing, and receive an income of zero. Individuals who agreed to teach an entrant receive their utility or disutility from that choice.

By separating the cooperation choice from the rest of the choices made by individuals, we can divide the game into one subgame in which cooperation occurs, and another in which it does not. The cooperation decision depends on the teacher’s expectations about what the entrant will do, as they cannot observe whether the entrant has chosen to become a productive worker or a rent-seeker: a teacher faces a marginal social expectation to cooperate, receiving an infinitesimal ϵ of utility from providing the necessary skills to an entrant, but they receive a disutility of α if they later discover that they cooperated with a rent-seeker (we assume that rent-seeking is visible at the end of the game). Thus, an individual’s cooperation choice does not depend on the individual’s own productivity, and so all individuals will make the same choice in stage 1.

All individuals receive linear utility from consumption and face a utility cost of rent-seeking relative to productive work in stage 2,^18^ which may differ depending on the occupation. Specifically, we assume that it is more costly to become a rent-seeker within the occupation associated with one’s own caste; Munshi and Rosenzweig (2016) demonstrate that sub-caste groups serve as a network of mutual insurance, and we assume that rent-seeking individuals who remain with their own caste are more likely to be found out as rent-seekers and to lose their reputation within the caste, thus running the risk of losing that insurance.^19^ We model this difference of rent-seeking costs in a reduced-form way: the utility cost is d if rent-seeking from the other caste’s occupation, and d+m if stealing from one’s own caste’s occupation, where m>0.

In each occupation, output of a productive worker is given by their ability z. As in Murphy et al. (1991), rent-seeking is predatory and involves stealing uniformly from productive individuals within a given occupation: if s percent of active individuals within an occupation are rent-seekers, a fraction τs of each worker’s output is stolen and divided evenly among the rent-seekers within that occupation. τ is an exogenous parameter of institutional quality, which measures how easy it is for rent-seekers to steal from the productive workers. We consider only symmetric equilibria across occupations, so the result will be identical in each occupation, and thus we drop caste subscripts when they are not needed.

We will later show in section 3.2 that entrants attempting and failing to enter the other caste’s occupation will be an off-equilibrium outcome; absent such failures of cooperation, a productive individual with skill z will receive consumption y(z)=(1−τs)z. Rent-seekers’ consumption does not depend on the rent-seeker’s skill:^20^ $y_{t}=\tau (1-s)E(z)$, where E(z) is the average skill of productive workers in that occupation. Therefore, utilities are as follows:

$$\;U(z)=y(z)=(1-\tau s)z\;U_{tc}=\tau (1-s)E(z)-d-m\;\;U_{tf}=\tau (1-s)E(z)-d$$

where U(z) represents the utility of a productive worker with skill z, $U_{tc}$ is the utility for individuals who rent-seek within their own caste, and $U_{tf}$ is the utility for those who rent-seek from the other caste.

**Figure 2.**
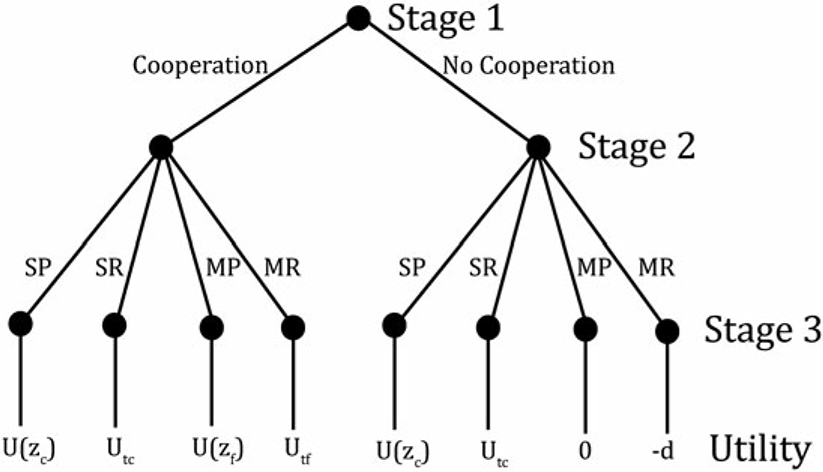
Game tree.

Figure 2 presents the timing and utility outcomes of our model in the form of a simple game tree.^21^ We introduce some new notation, denoting stage-2 choices with an S for “stay” or an M for “move” (i.e., move to a new occupation), followed by a P for “productive” or an R for “rent-seeking”. In stage 1, all individuals choose simultaneously, but as already mentioned the choice will be unanimous; then the choices at stage 2 and outcomes in stage 3 refer to a given individual.

### 3.2 Parametric assumptions

To simplify the analysis and intuition, we make a number of parametric assumptions and restrictions, which are summarized by the following assumption.

*Assumption 1.* We make the following assumptions in our model:

- d>−1, to ensure that it is efficient for everyone to work productively;
- there is a vanishingly small cost of switching occupations, an epsilon that can be ignored in welfare calculations but which ensures that ties are broken in favor of staying within the occupation associated with one’s caste;
- there is a similar vanishingly small cost of rent-seeking, which ensures that ties between producing and rent-seeking within an occupation are broken in favor of producing;
- τ<min{1+(d∕2),1}, to ensure that the return from rent-seeking is not so high as to cause highly-productive workers to steal;^22^
- m<τ, to rule out degenerate scenarios in which some individuals are willing to switch occupations and rent-seek even if this strategy produces zero income.

The combination of assumptions (ii) through (iv) ensures that types {2,1} and {2,2} will always stay in their own occupation and work productively, so that all future discussions of equilibrium allocations will focus on the choices of types {1,2} and {1,1}. Meanwhile, the vanishingly small cost of switching occupations implies that {1,1} types will never switch occupations to produce.

In a post-stage-1 subgame characterized by cooperation, we know that any action of SR is dominated by MR in a symmetric equilibrium because m>0. Meanwhile, for the {1,2} types, SP is dominated by MP, since productivity is higher in the other caste, and MR is also dominated by MP due to the assumption that τ<1+(d∕2). As a result, the only possible utility-maximizing choices in stage 2 are MP for type {1,2} and SP or MR for type {1,1}. Meanwhile, in a post-stage-1 subgame characterized by non-cooperation, we can easily see from the game tree that SP dominates MP (which produces zero utility), and SR dominates MR given our assumption (v) that τ>m, so that SP and SR are the only possible choices for both types {1,2} and {1,1}. In the following subsection, we use these results to characterize the equilibrium allocation.

### 3.3 Equilibrium solution

We now solve for the subgame perfect pure-strategy equilibrium of this game: one such unique equilibrium always exists, and by ruling out ties in utility with our tie-breaking assumptions, we abstract from mixed-strategy equilibria, which would only be relevant at the boundaries between different equilibrium regions in parameter space in any case. As mentioned above, the decision to cooperate with members of the other caste will always be unanimous: if $\hat{s}$ is the fraction of rent-seekers among individuals in an occupation who are from the opposite caste (and thus the probability that a teacher is matched with someone who will engage in rent-seeking), entry will be permitted if $\alpha \hat{s}>\epsilon$. Since ϵ is infinitesimally small, this means that in a pure-strategy equilibrium, cooperation will fail if the subgame equilibrium at stages 2 and 3 involves any types choosing MR.

We proceed by backwards induction, considering the subgame equilibria at stages 2 and 3 when cooperation is chosen in stage 1 and when it is not. Suppose, first of all, that cooperation is chosen in the first stage; then the two possible allocations are MP/MR and MP/SP, where the first action refers to {1,2} types and the second refers to {1,1} types. The equilibrium that maximizes total utility is MP/SP: both types work productively, making the pie as large as possible, and the {1,2} types who are more productive in the other caste’s occupation switch to make the best use of their skills. However, if the utility costs from rent-seeking are low enough, rent-seeking may occur in equilibrium; in particular, if d is small, the {1,1} types will want to switch occupations just for the sake of rent-seeking.

However, if some individuals choose to switch occupations for the purpose of rent-seeking, cooperation will fail in the first stage. Therefore, if parameters are such that MP/MR is the second-stage outcome in the presence of cooperation, cooperation is refused in the first stage, and occupational mobility is blocked: the {1,1} and {1,2} types both have a choice simply between SP and SR, and given that both types are in identical situations and break ties in favor of producing, the only possible equilibria are SP/SP and SR/SR. The former—an equilibrium in which every individual works productively within their own caste’s occupation—is inefficient because it does not allow for occupational mobility of the {1,2} types, but clearly SR/SR is even worse, as it generates an average output of 1 per person rather than the 1.5 that is produced in the SP/SP equilibrium.

Thus, there are three possible equilibrium outcomes: MP/SP, SP/SP, and SR/SR. For the efficient MP/SP outcome to be an equilibrium requires that the {1,1} types prefer SP over MR, or 1≥1.75τ−d. If this condition is not satisfied, the {1,1} types will prefer to switch occupations for the purpose of rent-seeking,^23^ which will be blocked in equilibrium. In that case, SP/SP will be an equilibrium if both types prefer SP over SR, which requires 1≥1.5τ−d−m, or τ≤(1+d+m)∕1.5. Clearly, (1+d+m)∕1.5>(1+d)∕1.75, which implies that the subgame perfect pure-strategy equilibrium can be described by the following proposition.

*Proposition 1.* The subgame perfect pure-strategy equilibrium to our model takes the following form:

- if τ≤(1+d)∕1.75, the equilibrium will be MP/SP;
- if τ∈((1+d)∕1.75,(1+d+m)∕1.5], the equilibrium will be SP/SP;
- if τ>(1+d+m)∕1.5, the equilibrium will be SR/SR.

**Figure 3.**
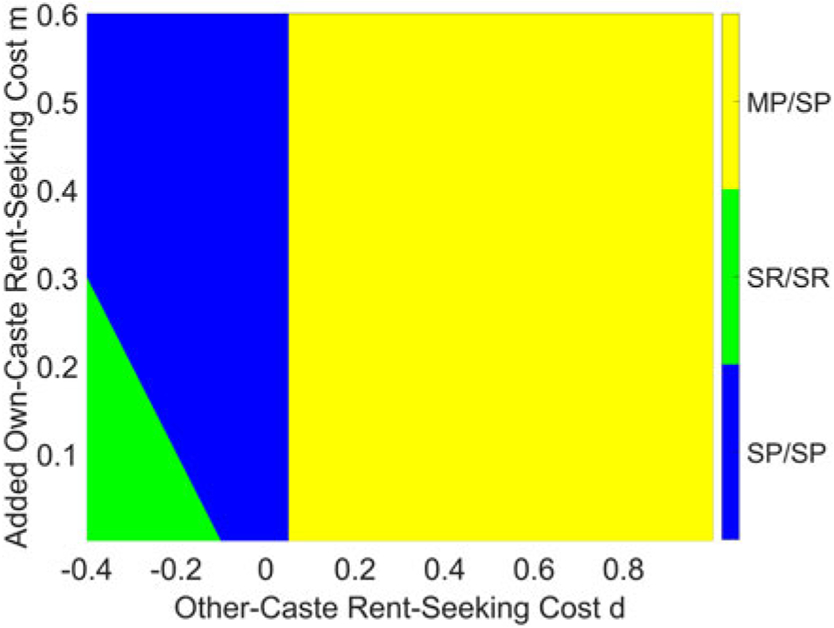
Equilibrium with τ=0.6.

**Figure 4.**
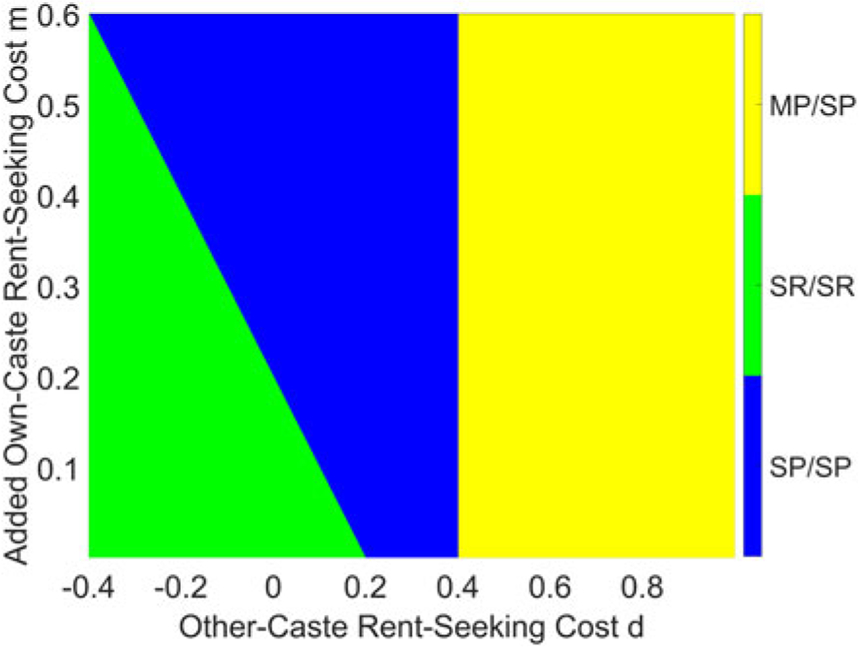
Equilibrium with τ=0.8.

This result can be seen graphically in Figure 3, which presents results with τ=0.6, and Figure 4, which presents the case where τ=0.8. Both figures demonstrate that, for a given τ, the good equilibrium (MP/SP) exists when d is sufficiently positive; if d is small, then the equilibrium depends on the value of d+m. Unsurprisingly, the good equilibrium is harder to reach when τ is large, in which case the monetary gain from rent-seeking is large.

The utility cost of rent-seeking across caste lines is d, and if this cost is small enough relative to the ease of rent-seeking τ, low-skilled individuals—those of type {1,1}—will want to switch occupations for the purpose of rent-seeking from the other caste. However, this threat of rent-seeking—indeed, the inability of low-skilled individuals to credibly commit not to rent-seek—will generate distrust between castes and an unwillingness to cooperate with people from other castes, leading to a breakdown of occupational mobility. If the utility cost of rent-seeking from one’s own caste m is not too large, this breakdown of occupational mobility can actually lead to more rent-seeking in equilibrium, in the SR/SR outcome. As noted earlier, this result of individual optimization is robust in a setting with rent-seeking from a team member within an occupation; this equivalence is demonstrated in Appendix D.

### 3.4. The role of caste

The model presented above represents a situation in which caste plays a meaningful role in society; our modelling of caste is limited to a horizontal differentiation between two *jatis*, but we present a setting in which casteism leads both potential rent-seekers and potential teachers to regard individuals differently depending on their caste origins. In our discrete-type model, it is not possible to have a single continuous variable for the strength of caste,^24^ but to connect our theoretical results to the stylized facts presented earlier, we can consider an alternative “caste-free” version of the model. In this version of the model, being of type c=1 or c=2 no longer indicates membership in a rigid social category, but simply indicates whether the individual is born to a parent that specialized in occupation 1 or 2. There are two substantive differences from the model presented above: because there are no inherent differences between the two groups, the utility cost of rent-seeking is constant regardless of which occupation an individual chooses to work in, so that m=0. Additionally, and more importantly, the first stage of the game vanishes: caste is no longer an observable characteristic, and thus cannot be used as a basis for choosing non-cooperation.

In this caste-free version of the model, there are only two possible equilibria: as above, if τ≤(1+d)∕1.75, the equilibrium will be MP/SP, whereas for any larger value of τ the equilibrium will be MP/SR.^25^ Thus, the comparison is simple: when caste is an important social force, occupational mobility ceases if τ>(1+d)∕1.75, and rent-seeking will also increase if τ>(1+d+m)∕1.5. Our model provides a microfoundation for the standard result that casteism generates occupational immobility: our results suggest that this could be due to the inability of individuals to commit not to steal from members of other castes, which leads to a lack of trust and a tendency to avoid economic interactions with other castes. This occupational immobility, meanwhile, can further explain a positive association between the strength of caste and rent-seeking: when casteism is sufficiently strong to prevent occupational immobility, in some cases individuals whose skills are a poor fit for their traditional occupation will find it more profitable to engage in rent-seeking, even if constrained to steal from their own caste.

## 4. Extensions & public policy

In the previous section, we presented our main model and showed how the resulting equilibrium depends on the relative costs and benefits of rent-seeking. In the current section, we present several extensions to our analysis. First, we add a new parameter to our model representing the utility cost of interactions with the other caste, which we interpret as a monetary or effort cost of interaction, such as that arising from the geographical distance between castes.^26^ The classic “contact hypothesis” of social psychology and sociology suggests that contact and interaction will improve interactions between groups in conflict [Allport (1954)]. We show, conversely, that interaction costs can actually increase the likelihood of attaining the good equilibrium, and we show that our empirical results are consistent with this interpretation. Second, we study the implications of extending the model to include individual education decisions, and show that our model predicts lower returns to education in the presence of strong forces of caste, which is also supported by supplementary empirical analysis. Finally, we analyze the effects of redistribution and demonstrate that certain types of redistribution can weaken incentives to rent-seek, thus encouraging occupational mobility and raising efficiency, though this effect depends on the degree of taxability of rent-seeking income.

### 4.1 Interaction costs

In this subsection, we introduce a new parameter b>0 to the model, which is a utility cost of entering the occupation associated with the other caste, whether for productive purposes or rent-seeking. The expected utility functions now take the following form (in the absence of failed entry):

$$\;U_{c}(z)=y(z)=(1-\tau s)z\;U_{f}(z)=y(z)-b=(1-\tau s)z-b\;\;U_{tc}=\tau (1-s)E(z)-d-m\;\;U_{tf}=\tau (1-s)E(z)-d-b$$

where $U_{c}(z)$ now represents the utility of a productive worker who stays in their own caste, while $U_{f}(z)$ is the utility of a producer who works in the other occupation.

**Figure 5.**
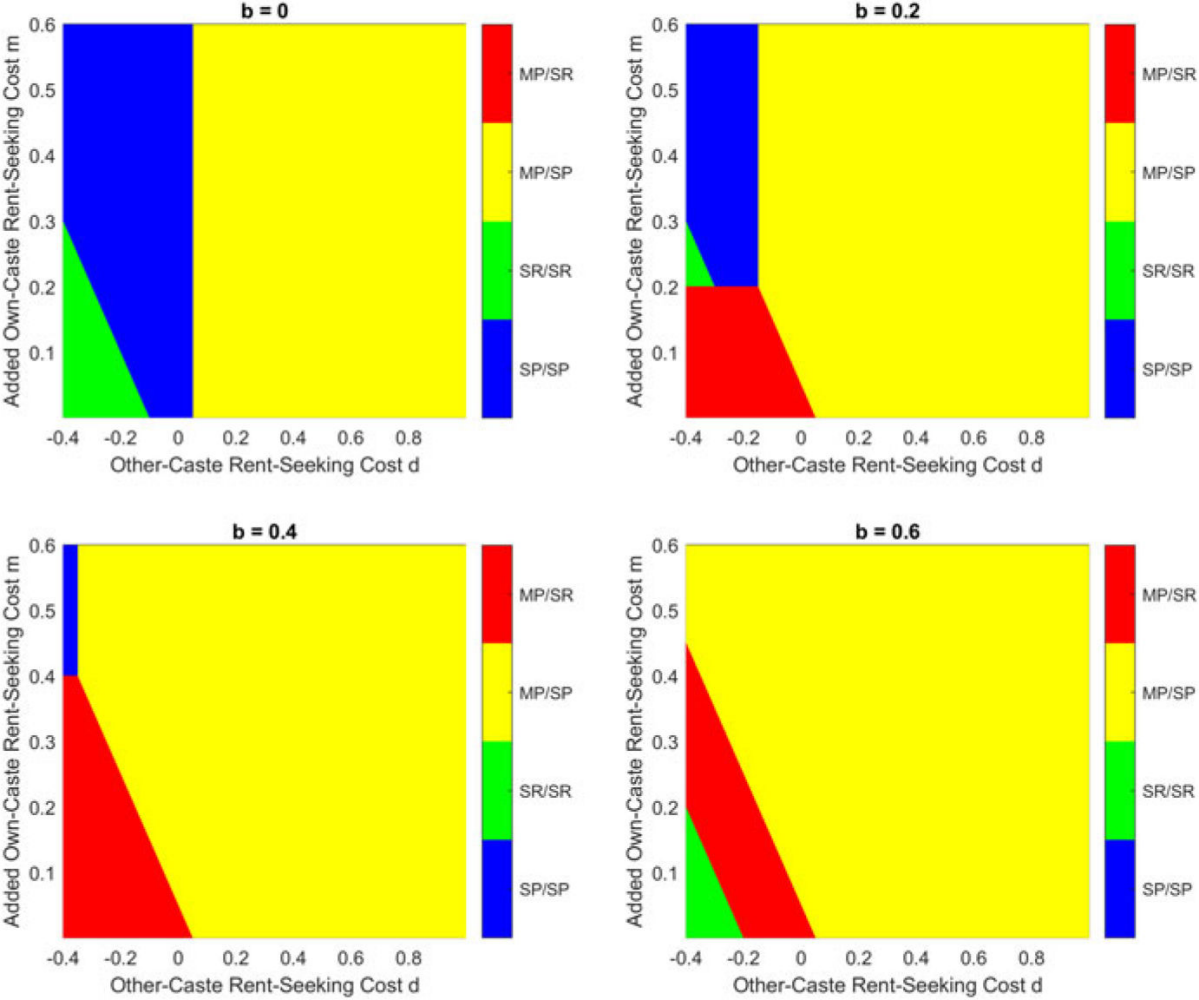
Equilibrium with τ=0.6.

**Figure 6.**
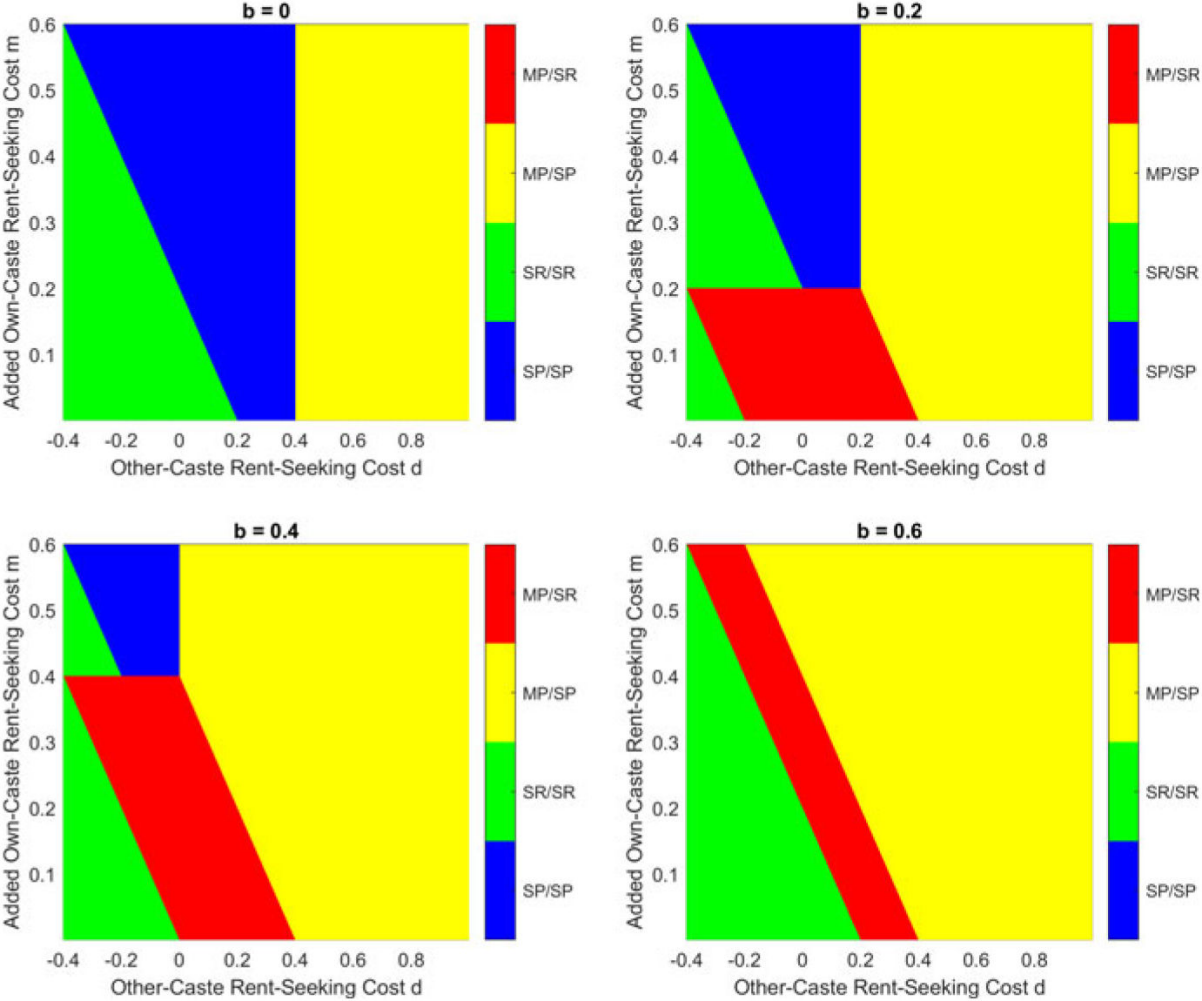
Equilibrium with τ=0.8.

In this setting, the equilibrium becomes significantly more complicated, and Appendix B presents the calculations, as well as the analytical results for equilibrium in Proposition 4. The equilibrium conditions are quite complicated, but the implications of introducing b can best be understood by considering the solution in graphical form: Figure 5 below presents the results with τ=0.6, while Figure 6 presents the case when τ=0.8. The most obvious result is that increasing b expands the region of parameter space that generates the good MP/SP equilibrium. A positive b also introduces a region in which MP/SR is the equilibrium, if m is smaller than b; and while it does not occur in the cases presented, when b is very large, it becomes possible that no pure-strategy equilibrium exists (which was not possible for b=0).

The main result of this section is that a positive b can actually improve the outcome in certain cases: given an equilibrium involving occupation-switching, a positive b reduces the average utility, but it increases the likelihood of attaining an efficient equilibrium with occupational mobility in the first place. The logic of this result is as follows: if it is fundamentally costly to interact economically with individuals from other castes, it is less likely that any given individual will want to switch occupations—and more importantly, the financial gain from switching would have to be substantial. In the current model, it is assumed that all rent-seekers obtain the same income (that is, rent-seeking skill is identical for all individuals), whereas there is a distribution of productive abilities, and thus only those with large gains from switching occupations will do so—that is, those who are the most productive in the other occupation. In a more general model with a continuous joint distribution of productive and rent-seeking skills, a similar result would apply if the distribution of productive skills was wider than that of rent-seeking skills.

Thus, with b>0, only those who really get paid from switching occupations are willing to tolerate the utility cost of doing so, and therefore cooperation becomes easier to sustain because it is more credible that occupation-switchers intend to work productively. If b is interpreted as a fundamental dislike of the other caste, this would generate the surprising result that mutual dislike between castes can, under certain circumstances, improve economic efficiency. However, the more interesting interpretation of b is as a measure of geographical integration: if individuals of different caste groups are closely clustered together, the cost of interaction b will be small.

This provides an explanation for why rent-seeking and occupational immobility are especially likely to happen in a country such as India: population density in India is extremely high, and members of another *jati* or *varna* are almost always nearby. Thus, the physical costs of interacting with other castes are low, which, in our model, makes it harder to reach the efficient equilibrium. Indeed, a test of this prediction in Appendix A provides empirical support, as presented in Table A.2: the association between local casteism and occupational immobility or social conflict is steeper in districts where population density is greater, where people of different *jati* are more likely to come into contact. For more detail, see Appendix A.

### 4.2 Education

**Figure 7.**
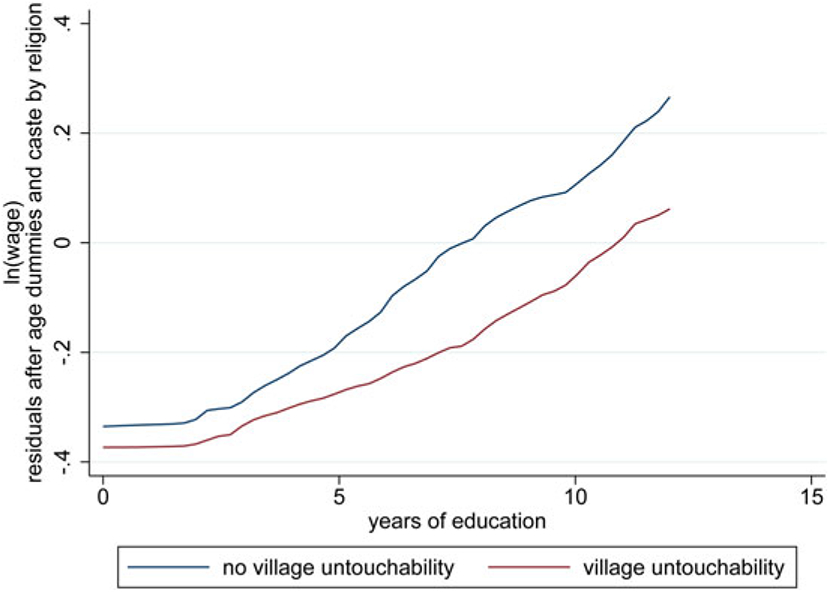
Returns to education.

We now introduce education as a choice variable. This is motivated by an additional stylized fact displayed in Figure 7, which uses the same IHDS data as section 2. The figure shows an interaction between education and untouchability: the return to education—visible in the gradient between education and log wages—is steeper for adult men in villages where casteism is low than in villages where casteism is high.^27^ Note that because the man’s own caste is controlled for, this interaction is a fact about the casteism of the neighbors in his village, not his own caste status or rank. As in our other empirical motivations, we interpret this result as an equilibrium outcome, not an effect of an exogenous force.

To model the relationship between casteism and education, we consider a case in which education e can be obtained in stage 2 at a cost $c(e)=(\eta ∕2)e^{2}$; to ensure well-defined boundaries of parameter space for equilibria, we assume that η<0.5 and d+m<(16∕9). Education is assumed to have no direct effect on the returns from rent-seeking, but it raises the output of a productive worker. Suppose that output for a productive individual with skill z and education e is ze, so that the income of such an individual is y(z,e)=(1−τs)ze; then the utility of this individual is:

$$U(z,e)=y(z,e)-c(e)=(1-\tau s)ze-\frac{\eta }{2}e^{2}.$$

Given a choice to be a productive worker, the first-order condition for e gives us $e^{⋆}(z)=((1-\tau s)z)∕\eta$, which implies that we can write indirect utility as:

$$U(z)\equiv U(z,e^{*}(z))=\frac{((1-\tau s)z{)}^{2}}{2\eta }.$$

Now consider the second stage of the game, if cooperation occurs and thus mobility is allowed in the first stage. As before, SR is dominated by MR given m>0, and for the {1,1} types, MP is dominated by SP given the tie-breaking rules; meanwhile, SP is dominated by MP for the {1,2} types. To ensure that MR is dominated by MP for the {1,2} types, and that MP is the dominant strategy for the {2,2} and {2,1} types, we modify our earlier assumption on τ: instead of part (iv) of Assumption 1, we now assume that $\tau <\text{min}\left\{6∕7,\left(4-\sqrt{4-6\eta d}\right)∕3\right\}$. Given this assumption, once again the only two possible second-stage pure-strategy outcomes are MP/SP and MP/MR. However, unlike in the baseline model, it is now possible that a region of parameter space exists in which no pure-strategy equilibrium exists, and thus we loosen our earlier assumptions—parts (ii) and (iii) of Assumption 1 on tie-breaking rules—to allow for mixed-strategy equilibria when no pure-strategy equilibrium exists. In particular, there may be a region in between MP/SP and MP/MR in which the {1,1} types randomize between SP and MR. However, cooperation will be denied in the first stage in the case of both the pure-strategy MP/MR equilibrium and the mixed-strategy case, and then occupational mobility will shut down as in section 3: MP is dominated by SP and MR is dominated by SR in the absence of cooperation.

MP/SP can be sustained as an equilibrium if τ≤(1+2ηd)∕6.5, as demonstrated in Appendix C. For any larger value of τ, cooperation fails and the possible outcomes are SP/SP, SR/SR, and a mixed-strategy equilibrium in which some of the {1,1} and {1,2} types rent-seek while the rest work productively; given that those two types are in identical situations when mobility is not possible, all that matters for describing the equilibrium is the overall fraction of rent-seekers, not the proportions in which the rent-seekers are drawn from each of the two types. Appendix C demonstrates that the outcome will be SP/SP when τ≤(1+2η(d+m)∕5, and SR/SR when $\tau >(10∕9)-\sqrt{\left(64∕81)-(8∕9)\eta (d+m\right)}$; in between the outcome will feature mixed strategies. The subgame perfect equilibrium can be described by the following proposition.

*Proposition 2.* The subgame perfect equilibrium to our model with education takes the following form:

- if τ≤(1+2ηd)∕6.5, the equilibrium will be MP/SP;
- if τ∈((1+2ηd)∕6.5,(1+2η(d+m))∕5], the equilibrium will be SP/SP;
- if $\tau \in ((1+2\eta (d+m))∕5,(10∕9)-\sqrt{\left(64∕81)-(8∕9)\eta (d+m\right)}]$, the equilibrium will feature types {1,1} and {1,2} mixing between SP and SR, with an overall proportion s of individuals engaged in rent-seeking that is described by 2η(d+m)=(1−τs)(5τ−1−τs);
- if $\tau >(10∕9)-\sqrt{\left(64∕81)-(8∕9)\eta (d+m\right)}$, the equilibrium will be SR/SR.

*Proof.* See Appendix C. □

Consider the effects of casteism on this equilibrium. Absent the ability of individuals to refuse to cooperate with members of the other caste, and with m=0, the equilibrium would feature MP/SP if τ≤(1+2ηd)∕6.5, MP/SR if τ was sufficiently large, and a mixed-strategy equilibrium in between. Our empirical analysis found that returns to education tend to be lower when caste is more important, and our results are consistent with that intuition: if the return to education is dy∕de=(1−τs)z, that return could be lower in the presence of caste for two reasons. First, if τ is sufficiently large, the equilibrium with caste will feature a higher level of rent-seeking as before, and a larger s will directly reduce the return to education. Second, in the absence of occupational mobility, productivity z will be lower for the {1,2} types, and a lower z will also translate into lower average returns to education.

If casteism reduces the returns to education, it would naturally also lead to lower values of education e, which suggests the possibility that casteism serves as a development trap: in the presence of caste-related rent-seeking, individuals will obtain less education, making the relative returns to a productive career lower and, under some circumstances, raising the relative attractiveness of rent-seeking.

### 4.3 Redistribution

As a final extension of our baseline model from section 3, we move to the question of public policy: what policy tools might help a government that desires to reduce rent-seeking and encourage occupational mobility? Obviously, a simple answer is to raise the utility costs of rent-seeking d and m, or to lower the return from rent-seeking τ, but this may be beyond the power of a government with limited institutional capacity, such as the government of India.^28^ Another equally—or even more—infeasible policy reform would be to eliminate the social force of caste, which has been a stated goal of the government of India for decades [Coffey and Spears (2017)].^29^

However, another possibility is the use of some form of redistributive policy. We suppose that the government has access to a proportional tax t<1 that can be applied to all sources of income, though perhaps less efficiently to income from rent-seeking: we assume that income from productive work is taxed at rate t, whereas income from rent-seeking is taxed at rate γt, so that γ≤1 is a “taxability” parameter for rent-seeking income. A classic income tax would presumably feature a low value of γ as income from predatory rent-seeking is likely to be illegal and would not be reported on an income tax return; however, a consumption tax might feature a γ at or close to 1 if all sources of income were indirectly subject to taxation at the time of consumption.

We assume that the proceeds from the tax are redistributed to the population via a lump-sum grant, and we continue to allow mixed strategies as in the previous subsection. We now replace part (iv) of Assumption 1 with τ<min{(2(1−t)+d)∕(2(1−γt)),1} in order to ensure that highly-productive individuals continue to work productively rather than rent-seek, and we also need to replace part (v) of Assumption 1 with m<(1−γt)τ, as otherwise type {1,1} will be willing to switch occupations even in the absence of cooperation, just to economize on rent-seeking costs. The conditions for equilibrium can then be described by the following proposition.

*Proposition 3.* The subgame perfect pure-strategy equilibrium to our model with proportional taxation takes the following form:

- if τ≤(1−t+d)∕(1.75(1−γt)), the equilibrium will be MP/SP;
- if τ∈((1−t+d)∕(1.75(1−γt)),(1−t+d+m)∕(1.5(1−γt))], the equilibrium will be SP/SP;
- if τ∈((1−t+d+m)∕(1.5(1−γt)), (1−t+d+m)∕(1.5−(γ+0.5)t))], the equilibrium will feature types {1,1} and {1,2} mixing between SP and SR, with an overall proportion s=(2.5τ(1−γt)−(1−t+d+m))∕(τ(1−γ)t) of individuals engaged in rent-seeking;
- if τ>(1−t+d+m)∕(1.5−(γ+0.5)t)], the equilibrium will be SR/SR.

*Proof.* For MP/SP to be an equilibrium requires that the {1,1} types prefer SP over MR, which requires (1−t)≥1.75(1−γt)τ−d, which simplifies to τ≤(1−t+d)∕(1.75(1−γt). If this condition is not satisfied, the {1,1} types will prefer MR, which will be prevented by a failure of cooperation in stage 1, and a failure of cooperation will shut down occupational mobility as before. SP/SP will then be the outcome if both types prefer SP to SR, which requires (1−t)≥1.5(1−γt)τ−d−m, or τ≤(1−t+d+m)∕(1.5(1−γt)), and SR/SR will be the outcome if both types prefer SR, which requires (1−γt)τ−d−m>(1−t)(1−0.5τ), or τ>(1−t+d+m)∕(1.5−(γ+0.5)t). For any values of τ in between these two, types {1,1} and {1,2} must mix at a rate that equalizes the returns from SP and SR, which gives (1−t)(1−τs)=(1−γt)τ(2.5−s)−d−m, which rearranges to give the expression above for s. □

As long as 1−t+d>0, an SP/SP region of parameter space exists, and the critical values are in the correct order: as τ increases from zero, it will pass through a region of MP/SP, followed by SP/SP, the mixed-strategy equilibrium, and SR/SR.

Aside from the mixed-strategy equilibrium, the structure of equilibrium is otherwise unchanged; however, the thresholds are affected by taxation. Consider the condition for an efficient MP/SP equilibrium: τ≤(1−t+d)∕(1.75(1−γt)). The right-hand side of this expression is increasing in t if and only if d>((1−γ)∕γ) : if rent-seeking is sufficiently costly in utility terms, and thus done for money and not for fun, redistribution reduces the financial gain from rent-seeking and thus the incentive to switch occupations in order to rent-seek; d must be greater than (1−γ)∕γ rather than zero to offset the direct positive effect of taxation on rent-seeking if γ<1. Thus, taxation could encourage cooperation, improving occupational mobility and raising efficiency, as long as that taxation does not itself encourage rent-seeking excessively through a low value of γ; note that if γ=1, as in the case of a perfect consumption tax, the condition for efficient taxation is weakened to d>0.

Similarly, consider the condition for the existence of some rent-seeking in equilibrium: τ>(1−t+d+m)∕(1.5(1−γt)). The right-hand side of this expression increases in t if and only if d+m>(1−γ)∕γ, which is a weaker condition than d>(1−γ)∕γ given that m>0.^30^ In this model, redistribution may not only raise occupational mobility; it may also reduce the incentive to engage in rent-seeking behavior, and both changes will tend to improve efficiency.

Thus, our results suggest that, as long as rent-seeking is sufficiently costly in utility terms—enough to outweigh any direct positive effect of taxation on rent-seeking caused by γ<1—taxation may encourage occupational mobility and the mingling of castes, leading to potential efficiency gains from taxation. In a more general model in which taxation also reduces labor effort on an intensive margin, this conclusion could be more ambiguous, but it at least introduces the possibility of a new efficiency motivation for redistribution in less-developed economies. Indeed, our results suggest that it is possible that high-income people may prefer higher levels of redistribution: if someone is going to come and take your money, better that it is the government which will directly transfer it to the poor to supplement their labor incomes, rather than it being stolen by individuals who have no other labor income and need a larger amount to get by. A further implication of our results is that the optimal level of taxation is likely to be increasing in casteism.

However, our results do vary significantly with the value of γ: if the tax available to the government is a labor income tax which cannot be applied to rent-seeking income, then γ=0 and a higher tax rate will always encourage more rent-seeking. This simply implies that it is very important to consider the form of redistribution used; consumption and other indirect forms of taxation may feature values of γ that are much closer to one, and indeed this may be a partial explanation for why income taxation represents a very small portion of the overall revenues of the Indian government: formal income taxation tends to discourage formal labor market participation, encouraging rent-seeking along with other less formal types of employment.

## 5. Conclusion

In this theoretical paper, we focus on three important features of modern-day India: caste, rent-seeking, and occupational immobility. We motivate our contribution with an empirical analysis based on data from the IHDS-II, from which we are able to produce a series of stylized facts indicating that, among other things, these three features of India are significantly positively correlated across space.

We then present a model that can explain these findings. If castes are associated with occupations and it is easier or less costly to rent-seek across caste lines, some people who want to switch to a different occupation do so to find an easier place to seek rents. This creates distrust between castes, and an unwillingness to cooperate with people from other castes, which shuts down the possibility of occupational mobility. Finally, if as a result, many workers are stuck in jobs that are a poor fit for their talents, rent-seeking may become even more attractive and prominent than it would in the absence of caste.

We continue our analysis of the implications of our model with a number of extensions: first, we find that it may be beneficial if it is costly to interact with other castes—which could explain why rent-seeking and immobility are particular problems in India, which has a high population density and in which it is never hard to find a member of another *jati*. Our model also conforms to an empirical result that returns to education are likely to be lower when caste is more important. Finally, we demonstrate that certain forms of redistribution may reduce rent-seeking and thus the segregation of castes, leading to a more efficient equilibrium.

Our model is a simple static model, but the reasoning applies in a dynamic sense as well: if new generations enter each period and choose a fixed occupation for the rest of their life, reaching a good equilibrium may be even harder than in a static model, for reasons similar to the model of collective reputations in Tirole (1996). Additionally, our model and the insights it generates could be applied to other contexts, in other developing countries, or even in a country such as the USA, where considerable debate in recent years has focused on the extent to which crime is committed within or between racial groups.^31^ We are hopeful that our paper will help to advance the literature on economic choices and group identities more broadly: social inequality can cause enduringly worse economic outcomes for everyone in equilibrium.

## Footnotes

1 Baland and Francois (2000) argue that resource booms can lead to an increase in unproductive rent-seeking, depending on the nature of the initial equilibrium, and Mehlum et al. (2006) find supporting evidence: countries with producer-friendly institutions benefit from increased resource revenues, while the opposite is true for countries with “grabber-friendly” institutions. Murphy et al. (1991) analyze the effect of rent-seeking on growth, where rent-seeking is predatory as in our paper.

2 See, e.g., the empirical debate between papers such as Persson and Tabellini (1994) and Forbes (2000), and theoretical analyses such as Galor and Moav (2004).

3 Casteism in India is an influential institution [Khamis et al. (2012)] that matters for many people: of the 1.3 billion people in India, essentially all of whom we argue are affected by the consequences of caste for India’s labour markets, over 200 million are Dalits (the lowest-ranking castes, formerly called “untouchables”), a disadvantaged population two-thirds as large as the USA or two-fifths as large as the EU.

4 Similarly, Kumar et al. (2002) find that, in 1971, 75% of male workers stayed in the same broad class of employment as their father, and that this measure of immobility had only declined to 71% by 1996. Reddy (2015) goes so far as to argue that, if one controls for changes in occupational structure, the probability of occupational mobility has actually declined in the last 30 years.

5 There are traditionally 4 *varna*: Brahmins, Kshatriyas, Vaishyas, and Shudras; the Dalits, or untouchables, are traditionally considered to be outside the caste system, and thus constitute a separate 5th layer. Typical estimates of the number of *jati* represented in India are on the order of 4,000: Johnson and Johnson (2017) states that “there are perhaps more than 3,000 jatis in India,” whereas Jalal (1995) claims that the estimated number of *jati* in the Vaishya and Shudra *varna* alone is between 3,000 and 4,000.

6 Furthermore, Akerlof (1976) does not generate predictions about policy implications, which will be an important extension of our paper.

7 In an empirical study of social distance in Sweden with survey data, Johansson-Stenman (2008) finds that “perceived trustworthiness appears to decrease generally with social distance.” We believe that casteism could generate, as an equilibrium outcome, an extreme version of this general social phenomenon.

8 On a similar note, Bros and Couttenier (2015) and Sharma (2015) find that certain interactions between castes in India are associated with higher rates of violent crime against lower castes.

9 As noted in Figure 1, for this graphical analysis, we first take residuals of this variable at the household level, after controlling for state fixed effects and own caste and religion.

10 Although this stylized fact is popularly understood within India to be correlated with the difference between northern plains states such as Uttar Pradesh, and southern states such as Kerala or mountain regions, in our supplementary Appendix we show that this is true even for variation *within* Indian states.

11 An alternative would be to study political or bureaucratic corruption, but our model is about social inequality and economic markets; it has no state or government agents.

12 Acemoglu (1995) is another example of a single-sector model with an option of uniform predatory rent-seeking, while Mehlum et al. (2005) model a two-sector economy in which predatory rent-seeking occurs within a “modern” sector but not in a “subsistence” sector; in the latter paper, rent-seeking is based on a form of directed search rather than being uniform.

13 See, e.g., the discussion of the occupational aspects of the caste system in Hnatkovska et al. (2013).

14 Our focus on *jati* is consistent with the findings, empirical and theoretical, in Mayoral and Ray (2016) that conflict between social groups over private prizes is more likely to take place at the level of smaller groups. Any rent-seeking that takes place between *jati* at different levels of the hierarchy could also be captured by our model, but we do not attempt to model all forms of rent-seeking in India.

15 In Appendix A, we also report results using two alternative measures of caste attitudes and practices: knowing of intercaste marriage in the community, and belonging to a caste association. Results of the former are inconsistent, whereas results of the latter are closer to our main results; however, as discussed in the Appendix, we think that these variables are better understood as equilibrium caste practices rather than the casteist attitudes that we want to measure.

16 Essentially, we hold fixed other sources and types of rent-seeking, which are implicitly included within our definitions of output and utility, which can be considered as after-other-rent-seeking output and utility. As pointed out by an anonymous referee, even when considered rent-seeking across caste lines, one might expect a significant fraction of rent-seeking to occur between caste groups at different levels of the hierarchy rather than between horizontally-equivalent *jati*; as we abstract from hierarchy, we cannot differentiate these different types of rent-seeking.

17 In practice, rent-seeking could take a variety of forms, especially as permitted by incomplete information about effort and products. Somebody working for a member of another caste could put in less effort in a wage-earning job in a difficult to observe way, or could produce a lower-quality (or no-quality) product despite collecting pay. Development economists have long recognized the information challenges in contracting for *agricultural* labor, where (in part because workers may be physically spread out from managers) effort is particularly difficult to observe [Ray (1998)].

18 We normalize the disutility of productive work to zero.

19 We assume that the utility cost does not depend on the caste identity of the occupation’s membership; in equilibrium it will always be the case that at least 50% of the workers in an occupation are from the caste associated with that occupation.

20 Our results would be unchanged with a distribution of rent-seeker skill if individuals were uninformed about their own skill prior to choosing their action. More generally, our intuition is based on the idea that there is a narrower distribution of rent-seeking skill relative to productive skill; we could construct a more general model with such an assumption that arrives at similar results.

21 We do not include in the game tree any disutility from agreeing to teach an entrant who becomes a rent-seeker, which will not happen in equilibrium.

22 If this condition on τ is not satisfied, equilibrium may require a mixed strategy in which some individuals with a skill of 2 engage in rent-seeking.

23 It is easy to show that $\tau^{⋆}=(1+d)∕1.75$ partitions the space of potential outcomes if cooperation occurs: MP/SP is the outcome if τ≤(1+d)∕1.75, whereas MP/MR is the result if τ>(1+d)∕1.75.

24 For such continuity to be meaningful, there would need to be some continuity in the information available when an individual decides whether or not to cooperate. For example, with partial observability of types, where a probability that an individual is of type {1,1} is visible at the time of the cooperation decision, the value of α would determine the fraction of entrants that are allowed to enter the opposite occupation if ϵ was finitely positive.

25 Given that ties between moving and staying within one’s “occupation at birth” are broken in favour of staying, SR will be chosen over MR when m=0.

26 Alternative interpretations of this parameter are (i) that it measures the extent to which castes fundamentally dislike each other (though the utility cost is borne only by the occupation-switcher), or (ii) the extent to which the occupations of the castes are functionally distant from each other.

27 In particular, the figure plots locally weighted regressions, for observations which are individual adult men, of the log of the equivalent hourly wage in rupees on years of education, where wages are residualized after regression on indicator variables for age, the man’s caste category, and the man’s religious category.

28 Baumol (1990) discusses the possibility of policy to discourage or encourage rent-seeking behaviour, and argues that at various places and times in human history, rent-seeking has often been seen as not only normal, but actually virtuous. Given the ongoing campaigns for reservations in public employment by a wide variety of groups in India—jobs which are often associated with benefits from rent-seeking—this may not be an inaccurate model of India today.

29 Although it is outside the scope of our model, casteism could influence both the goals and the capabilities of the state and its bureaucracy to achieve redistribution and other purposes [Lamba and Spears (2013), Singh (2015)], as well as human capital through sanitation and early-life infectious disease [Gupta et al. (2016), Duh and Spears (2017)].

30 The condition for SR/SR has a similar critical value, becoming less likely to be satisfied as t increases if and only if d+m>(1−γ)∕γ+0.5, which is an even weaker condition than that for non-zero rent-seeking.

31 See, for example, Lee (November 25, 2014).

## Appendix A

### Empirics of stylized facts

The main contribution of this paper is its theoretical model, which can account for the stylized facts presented in section 2. Our claim is that these observed correlations are equilibrium outcomes: casteism, occupational immobility, low income, and rent-seeking mutually cause one another in equilibrium. We do not claim that we have an exogenous source of variation in casteism that we exploit to identify a causal effect of one upon the other.

Instead, what is novel about our empirical results is that we are able to document correlates of *casteism*, the disposition towards and practice of social differentiation, rather than a household’s own caste identity, or the caste composition of the households in a village.^32^ This is because we use a novel survey question^33^ included in the 2012 round of the IHDS, which asked 27,000 rural households about households’ practice of untouchability, meaning whether or not they enforce the rules of untouchability in their interactions with Dalit (very low caste-ranking) people.^34^ The survey asked two questions to each household-level respondent:

- A: In your household do some members practice untouchability?
- B, *if no to A*: Would there be a problem if someone who is scheduled caste were to enter your kitchen or share utensils?

In general, we count a household as practicing untouchability if it answers yes to A or B; we then compute village-level casteism as the average over this household-level variable in the village. This question is a valuable step beyond what is ordinarily included in survey datasets such as the National Sample Survey or the Demographic and Health Surveys because it offers a measure of social attitudes of the sort that we study.^35^ In two specifications, for robustness, we instrument for our casteism measurement, as a response to measurement error. Casteism is a complex object: not only do we have a simple measurement of it, but it is also only measured for a sample of households, which implies that we have a measurement-with-random-sampling-error of the true village-level average. Measurement error may be especially important in our district fixed effects regressions because about a quarter of the variation in our casteism measure is explained across districts (which reflects the correlation of caste with geography across India).

We construct geographic averages of our dependent and independent variables to represent the fact that our model is a model of an equilibrium in a local labor market. We also construct village-average control variables, to rule out that our results are driven by patterns of the composition of the village. In particular, we regress

(1)
$${\bar{outcome}}_{vs}=\beta {\bar{casteism}}_{vs}+\delta_{s}+\Gamma C_{vs}+f(X_{vs})+\varepsilon_{vs},$$

where v indexes villages, s indexes states, β is the coefficient of interest, δ are state fixed effects, C is a vector of controls for the caste (6 categories) and religion (9 categories) composition of the village, and X is a set of extended controls that will be included for robustness. Because villages are primary sampling units of the rural IHDS, village averages are consistent for village means.

Occupational mobility is categorized for each household head based on the responses to two survey questions:

- What is the principal source of income for the household?
- What was the primary occupation of the household head’s father (for most of his life)?

Table A.1 presents regression results for each of the five outcome variables reported in Figure 1. Panel A reports a direct implementation of a regression equation (1), with state fixed effects. Although there is considerable variation in casteism across the states of India, these state fixed effects ensure that our results are not merely a spurious reflection of geography or variation in policy or governance. Panels C and D substitute district fixed effects for state fixed effects; districts in India are approximately 600 administrative sub-divisions of states. These district fixed effects, therefore, account for a fine degree of variation in geographic and policy environments. Panel D further adds controls for the education of household heads (16 indicators for possible levels of schooling) and controls for the occupational category of household heads’ fathers, as used to construct the occupational mobility variable. These ensure that the particular human capital or occupation of households (e.g., farming) is not driving our results about the *match* of occupational category.

Panel B replaces the OLS framework of (1) with an IV approach that instruments for the village average of “A” practice of untouchability with the village average of “A or B” practice of untouchability. This instrumentation is motivated by the possibility of measurement error in survey-reported untouchability, both because respondents may not answer correctly for their entire household and because within-village information is computed from a sample survey. Panel E uses Panel B’s instrument as well as a further instrument: the fraction of households that report in a survey question they are aware of intercaste marriage in their community. We thank an anonymous referee for this suggestion, but we also wish to note an ambiguity: the question asks about intercaste marriage in the respondent’s “community,” which may be understood in this context as a social community—indeed a caste group—rather than a *local* community, which is the focus of our model and the unit of analysis in our empirics.

The principal message of Table A.1 is that these variations in specification, dependent variable, and functional form mutually cohere. The purpose of this analysis is not to estimate a causal effect. Rather, we document these this robust, coherent set of correlations as evidence for a stylized fact that motivates our main theoretical contribution.

We also emphasize the economic magnitude of these coefficients. In the IHDS, 3.8% of household respondents report a theft, and 1.8% reports an attack, so these are large differences. In the average village, about 5% of respondents report an attack or theft, so a linear coefficient of 4 percentage points is economically large relative to the magnitude of this variable. Similarly, only 13% of respondents report a “lot of conflict” so point estimates around 20 percentage points—or even the non-statistically-significant estimates of 6–8 percentage points in panels C through E—are a large variation in conflict. Rent-seeking and casteism do not merely correlate: they substantially covary across the variation of conflict that exists.

As an extension, Table A.2 tests the implication of section 4.1: where interaction costs are low, the pernicious effects of casteism should be more severe. We operationalize low interaction costs with population density: population density is very high even in rural India, in international comparison, and members of different castes often live in the same political and economic villages. We measure population density finely at the district level. Notice that this prediction runs counter to other well-known theories in the social science literature, such as Allport’s (1954) “contact hypothesis,” prominent in sociology and social psychology, which hypothesizes that interpersonal contact reduces prejudice and discrimination between group members.

Table A.2 reports simple interactions between village casteism and district population density, predicting the same dependent variables as in Table A.1. As a robustness check, we add a sixth dependent variable, which is the first principal component of the other five; note that the sign on log income, appropriately, is the only of the five that is negative in the principal component analysis. We see the interaction in the predicted direction: consistent with our model, but against the “contact hypothesis,” local casteism is more steeply associated with occupational immobility and social conflict where population density is greater.

A final piece of empirical evidence, in Table A.3, investigates two alternative measures of caste attitudes and practices.^36^ Neither of these quite captures the same thing as *casteism*—in part because they measure equilibrium behaviors, rather than casteist attitudes—but we study them for a richer understanding of the complexities of measuring caste. Yet, neither alternative turns out to be meaningfully correlated with reported casteism. The alternative measure in Panel A is the fraction of surveyed respondents reporting belonging to a caste association. It is correlated with the village being richer, but also with it seeing more theft, threats, and conflict. The alternative measure in Panel B is the fraction of respondents who report knowing of intercaste marriage in their “community;” as we discussed above, interpreting this variable is complicated by the fact that, to some respondents and surveyors, “community” probably did not mean local village. Unsurprisingly, the results are inconsistent. This coheres with the possible multiple meanings of “community” in this context (i.e., not villages).

**Table A.1.** Robustness of stylized facts: regression with alternative specifications

Dependent variable:	(1) Match father’s job	(2) ln(income p.c.)	(3) Geographic immob.	(4) Theft or threat	(5) Village conflict
*Panel A: State fixed effects, own caste and religion controls*					
Casteism	0.0871**	−0.472*	0.0819***	0.0418*	0.191***
	(0.0419)	(0.259)	(0.0221)	(0.0234)	(0.0616)
*n* (villages)	1,420	1,419	1,420	1,420	1,420
*Panel B: State fixed effects, IV, own caste and religion controls*					
Casteism	0.105**	−0.571*	0.0992***	0.0505*	0.231***
	(0.0495)	(0.297)	(0.0262)	(0.0279)	(0.0734)
*n* (villages)	1,420	1,419	1,420	1,420	1,420
Underid test	57.58	58.12	57.58	57.58	57.58
Weak id test	612.7	613.6	612.7	612.7	612.7
*Panel C: District fixed effects, own caste and religion controls*					
Casteism	0.104**	−0.393**	0.0744***	0.0381*	0.0555
	(0.0433)	(0.165)	(0.0238)	(0.0213)	(0.0593)
*n* (villages)	1,420	1,419	1,420	1,420	1,420
*Panel D: District fixed effects, own caste and religion and extended controls*					
Casteism	0.117***	−0.447***	0.0785***	0.0404*	0.0655
	(0.0416)	(0.162)	(0.0245)	(0.0207)	(0.0589)
*n* (villages)	1,420	1,419	1,420	1,420	1,420
*Panel E: District fixed effects, two instruments, own caste and religion and extended controls*					
Casteism	0.135***	−0.529***	0.0921***	0.0509**	0.0844
	(0.0491)	(0.190)	(0.0289)	(0.0247)	(0.0709)
*n* (villages)	1,396	1,395	1,396	1,396	1,396
Underid test	104.4	105.1	104.4	104.4	104.4
Weak id test	607.7	606.6	607.7	607.7	607.7

*Note*: Data are from the 2012 India Human Development Survey. The survey is a household-level survey; we have computed village level averages by collapsing household-level data into means. In Panel B, instrumentation is used to address measurement error in casteism: the fraction of households reporting a more expansive definition of practicing casteism is used to instrument for the fraction of households reporting a less expansive definition. In Panel E, both this instrument and the fraction of households that report knowing of intercaste marriage in their community are used. Elsewhere, the more expansive definition is used. All panels control for the caste and religion composition of the village; panels D and E adds extended controls for the composition of the village by the household head’s education and by the occupational category of the household head’s father. Standard errors are robust but not clustered, because they are already collapsed by survey primary sampling unit.

The “underid test” is the Kleibergen-Paap $\chi^{2}$ test statistic for underidentification. The “weak id test” is the Kleibergen-Paap *F*-test for weak identification.

Two-sided *p*-values:

* *p* < 0.10

** *p* < 0.05

*** *p* < 0.01.

**Table A.2.** Extension of stylized facts: Interaction of casteism with population density

Dependent variable:	(1) Principal comp.	(2) Match father’s job	(3) ln(income p.c.)	(4) Geographic immob.	(5) Theft or threat	(6) Village conflict
Casteism	1.429***	0.0152	−0.524***	0.143***	0.0497***	0.216***
	(0.316)	(0.0392)	(0.202)	(0.0238)	(0.0151)	(0.0623)
Population density	−0.000226**	−0.0000869***	−0.000101	0.00000251	−0.00000288	−0.000156***
	(0.000111)	(0.0000325)	(0.0000727)	(0.0000135)	(0.00000735)	(0.0000552)
Casteism × density	0.00153***	0.000158*	0.000107	0.0000387	0.000167***	0.000459***
	(0.000520)	(0.0000841)	(0.000307)	(0.0000240)	(0.0000514)	(0.000136)
*n* (villages)	1,419	1,420	1,419	1,420	1,420	1,420

*Note*: Data are from the 2012 India Human Development Survey. The survey is a household-level survey; we have computed village level averages by collapsing household-level data into means. Standard errors are robust but not clustered, because they are already collapsed by survey primary sampling unit. District-level population density is demeaned to preserve the interpretation of the interaction. A constant term is omitted from the table; no further controls are included in these regressions.

Two-sided *p*-values:

* *p* < 0.10

** *p* < 0.05

*** *p* < 0.01.

In short, in some specifications of Table A.3 these alternative approaches are consistent with our model, but not all: the mere fact that they are not correlated with reported casteism itself cautions against their use as a substitute. These variables are about behaviors, not attitudes, and whether attitudes are revealed in behaviors depends on the equilibrium costs and benefits of doing so. So, we find that the results in Table A.3 suggest (as studies of discrimination have found in other contexts) that the way of measuring caste prejudice matters; noting this can reinforce the unique value of the IHDS having asked the novel question on caste attitudes that do not exist in other data sources and that we are able to study.

## Appendix B

### Proof of equilibrium with b>0

In the model presented in section 4.1, the branch of the game tree in which cooperation fails and thus mobility is blocked is unaffected when we introduce a positive utility cost of cross-caste interaction b, since no such interaction will take place in that case. However, the outcome when cooperation takes place is far more complicated than before. Consider the possible actions of the {1,2} and {1,1} types; as before, the {2,2} and {2,1} types always choose SP.

The {1,2} types have a choice between MP, SP and SR; type-2 individuals will always work productively, given the assumption that τ<1+(d∕2), which rules out MR. Meanwhile, the {1,1} types have a choice between MR, SP and SR, as MP is always dominated by SP for these individuals. SP/SR and SR/SP are impossible given the tie-breaking rules, but we cannot rule out any other combinations without considering the relative values of b and the other parameters; this leaves a set of 7 possibilities for equilibrium, which depend on the following conditions:

1. MP/MR: we need that type {1,2} prefers MP to SP and SR, and that type {1,1} prefers MR to SP and SR; the condition that type {1,1} prefers MR to SR, along with the condition that τ<1+(d∕2), implies that the condition that type {1,2} prefers MP to SR is satisfied, and thus the conditions for MP/MR to be an equilibrium are:
  b<1−0.25τ&1<1.75τ−d−b&b<m.
2. MP/SP: we need that type {1,2} prefers MP to SP and SR, and that type {1,1} prefers SP to MR and SR; the condition that type {1,2} prefers MP to SP, along with the condition that type {1,1} prefers SP to SR, implies that the condition that type {1,2} prefers MP to SR is satisfied, and thus the conditions for MP/SP to be an equilibrium are:
  b<1&1≥1.75τ−d−m&1≥1.75τ−d−b.
3. MP/SR: we need that type {1,2} prefers MP to SP and SR, and that type {1,1} prefers SR to MR and SP; the condition that type {1,2} prefers MP to SR, along with the condition that type {1,1} prefers SR to SP, implies that the condition that type {1,2} prefers MP to SP is satisfied, and thus the conditions for MP/SR to be an equilibrium are:
  b<2(1−τ)+d+m&1<1.75τ−d−m&b≥m.
4. SP/MR: we need that type {1,2} prefers SP to MP and SR, and that type {1,1} prefers MR to SP and SR; the condition that type {1,1} prefers MR to SP, along with the condition that type {1,2} prefers SP to SR, implies that the condition that type {1,1} prefers MR to SR is satisfied, and thus the conditions for SP/MR to be an equilibrium are:
  1≥1.5τ−d−m&b≥1−0.25τ&1<1.5τ−d−b.
5. SP/SP: we need that type {1,2} prefers SP to MP, that type {1,1} prefers SP to MR, and that both types prefer SP to SR; the conditions for SP/SP to be an equilibrium are:
  b≥1&1≥1.5τ−d−b&1≥1.5τ−d−m.
6. SR/MR: we need that type {1,2} prefers SR to MP and SP, and that type {1,1} prefers MR to SP and SR; the condition that type {1,1} prefers MR to SR, along with the condition that type {1,2} prefers SR to SP, implies that the condition that type {1,1} prefers MR to SP is satisfied, and thus the conditions for SR/MR to be an equilibrium are:
  1<1.5τ−d−m&b≥2(1−τ)+d+m&b<m.
7. SR/SR: we need that type {1,2} prefers SR to MP, that type {1,1} prefers SR to MR, and that both types prefer SR to SP; the conditions for SR/SR to be an equilibrium are:
  b≥m&1<1.5τ−d−m&b≥2(1−τ)+d+m.

**Table A.3.** Correlation of alternative measures of caste attitudes and practices with variables in Table A.1

Dependent variable:	(1) Casteism A	(2) Casteism B	(3) Match father’s job	(4) ln(income p.c.)	(5) Geographic immob.	(6) Theft or threat	(7) village conflict
*Panel A: Fraction of PSU reporting belonging to a caste association*							
Caste association	0.0277	0.0221	0.120	0.441***	0.0262	0.104**	0.230***
Membership	(0.0863)	(0.0743)	(0.0739)	(0.137)	(0.0353)	(0.0405)	(0.0800)
*Panel B: Fraction of PSU knowing of “community” intercaste marriage as independent variable*							
Intercaste marriage	0.109	0.100	−0.0623	−0.167	−0.0580*	0.0535***	0.0177
In community	(0.0685)	(0.0674)	(0.0499)	(0.200)	(0.0346)	(0.0181)	(0.0717)
*n* (villages)	1,417	1,417	1,417	1,416	1,417	1,417	1,417

*Note*: Data are from the 2012 India Human Development Survey and match Table A.1. Regressions are the same specification as Panel A of Table A.1 except with changed independent variable of interest. Two-sided *p*-values:

* *p* < 0.10

** *p* < 0.05

*** *p* < 0.01. As noted in the text, the survey question is ambiguous about whether “community” (in the Panel B question) refers to a local village (which is the unit we study) or a social or caste group (which is common usage in India).

It is easy to verify that the equilibrium of this subgame, when it exists, is always unique; however, as we will see, it is possible that a pure-strategy equilibrium does not exist when b is sufficiently large. In the latter case, mixed-strategy equilibria will exist if the utility-tie-breaking rules presented in Assumption 1 are dropped.

Any case in which MR is an outcome chosen by one of the types—that is, cases 1, 4, and 6—will be blocked in the first stage, as $\hat{s}$ and cooperation will fail, and in that case, the outcome is exactly as in section 3: SP/SP will be the outcome if τ≤(1+d+m)∕1.5, and otherwise the result will be SR/SR. Therefore, the subgame perfect pure-strategy equilibrium can be described by the following proposition.

*Proposition 4.* The subgame perfect pure-strategy equilibrium to the extended model with b>0 takes the following form:

- if b<1 and 1≥1.75τ−d−m and 1≥1.75τ−d−b, the equilibrium will be MP/SP;
- if b<2(1−τ)+d+m and 1<1.75τ−d−m and b≥m, the equilibrium will be MP/SR;
- if b≥1 and 1≥1.5τ−d−b and 1≥1.5τ−d−m, or if 1≥1.5τ−d−m and b≥1−0.25τ and 1<1.5τ−d−b, or if b<1−0.25τ and 1<1.75τ−d−b and b<m and τ≤(1+d+m)∕1.5, the equilibrium will be SP/SP;
- if b≥m and 1<1.5τ−d−m and b≥2(1−τ)+d+m, or if 1<1.5τ−d−m and b≥2(1−τ)+d+m and b<m, or if b<1−0.25τ and 1<1.75τ−d−b and b<m and τ>(1+d+m)∕1.5, the equilibrium will be SR/SR;
- if none of the above conditions is satisfied, there will be no pure-strategy equilibrium.

This complicated set of conditions is most easily represented graphically, and the equilibria for τ=0.6 and τ=0.8 can be found in Figures 5 and 6 in section 4.1.

## Appendix C

### Proof of equilibrium with education

We begin with the second-stage result if cooperation occurs in the first stage. MP/SP will be the outcome as long as type {1,1} prefers SP to MR, which requires:

$$\frac{1}{2\eta }\geq \frac{3.25\tau }{\eta }-d$$

and this simplifies to τ≤(1+2ηd)∕6.5. If τ takes a larger value, the resulting outcome will either be MP/MR or a mixed-strategy outcome, both of which will be blocked in the first stage.

If cooperation fails in the first stage, it is easy to show that, as in section 3, occupational mobility shuts down as neither type {1,1} or {1,2} wish to switch occupations for any reason. Therefore, SP and SR are the only possible choices for those types. Having dropped the tie-breaking rules, we need to consider three possible scenarios: SP/SP, SR/SR, and a mixed-strategy equilibrium in which types {1,1} and {1,2} mix between SP and SR. The outcome will be SP/SP if types {1,1} and {1,2} prefer SP to SR, which requires:

$$\frac{1}{2\eta }\geq \frac{2.5\tau }{\eta }-d-m$$

and this simplifies to τ≤(1+2η(d+m))∕5; as in the baseline case, (1+2η(d+m))∕5>(1+2ηd)∕6.5, and so an SP/SP region of parameter space always exists. Meanwhile, the outcome will be SR/SR if types {1,1} and {1,2} prefer SR to SP, which requires:

$$\frac{(1-0.5\tau {)}^{2}}{2\eta }<\frac{2\tau (1-0.5\tau )}{\eta }-d-m$$

and this simplifies to 2η(d+m)<(1−0.5τ)(4.5τ−1); we then use the quadratic formula to solve for $\tau >(10∕9)-\sqrt{\left(64∕81)-(8∕9)\eta (d+m\right)}$.

The critical minimum value of τ for SR/SR is always larger than the critical maximum value for SP/SP, and in between, the equilibrium will feature mixed strategies: some of the types {1,1} and/or {1,2} will work productively, and others will engage in rent-seeking. In particular, the overall proportion s of individuals engaged in rent-seeking needs to ensure that the utility from rent-seeking equals the utility from productive work, which implies:

$$\frac{(1-\tau s{)}^{2}}{2\eta }=\tau (1-s)E(ze)-d-m.$$

E(ze) can be written as $\left((1-\tau s)∕\eta )E(z^{2}\right)$, and $E(z^{2})=(2.5-s)∕(1-s)$, which allows us to write the above equation as:

$$\frac{(1-\tau s{)}^{2}}{2\eta }=\tau (2.5-s)\frac{1-\tau s}{\eta }-d-m$$

and this further simplifies to:

2η(d+m)=(1−τs)(5τ−1−τs).

We could further use the quadratic formula to solve for a closed-form solution for s, but the resulting expression does not add any new intuition to the results.

## Appendix D

### Model with rent-seeking as theft from teammates

This Appendix presents an earlier version of our model, in which predatory rent-seeking involves stealing from one team member rather than uniformly from the entire occupation. As we will demonstrate, the results from section 3 apply unchanged to this alternative version of the model.^37^ The results from all of the extensions in section 4 are also unchanged, except that a new mixed equilibrium occurs in the model with education: for $\tau \in ((1+2\eta d)∕6.5,\hat{\tau }]$, where $\hat{\tau }$ is a value in between (1+2ηd)∕6.5 and (1+2η(d+m))∕5, the equilibrium in Proposition 2 will feature type {1,2} choosing MP and type {1,1} mixing between SP and MR rather than a simple SP/SP equilibrium, with an overall proportion s of individuals engaged in rent-seeking that is described by (1−τs)(3.25τ−0.5τs−0.5)=ηd.

The basic context of the model is unchanged: there are still 2 horizontally-differentiated castes, where each is associated with an occupation, and the distribution of productivity types is the same as before. Each individual still chooses the occupation they want to work in, and whether to work productively or engage in rent-seeking, but then in stage 3, each individual within an occupation is randomly matched with one other individual within the same occupation in a production team. Rent-seeking is modelled as stealing from the teammate’s output, but productive individuals who are working in their own caste’s occupation and are matched with a member of the other caste then have a further choice between working cooperatively or uncooperatively, where uncooperative work produces output that is smaller but safe from rent-seeking.^38^ The structure of the game tree is otherwise unchanged: the cooperation decision is made in stage 1, the decision to engage in productive work or rent-seeking in stage 2, and the results are revealed in stage 3. Once again, the cooperation decision in stage 1 is unanimous.

The structure of output, income, and utility is the same as in the main model of the paper, except that a cooperative productive worker still produces z units of output, whereas an uncooperative worker only produces χz, where χ<1. If a worker is cooperative and their teammate engages in rent-seeking, the latter will steal τ percent of the former’s output; if a worker is uncooperative, a rent-seeking teammate will fail to steal any output and will receive an income of zero. If both teammates engage in rent-seeking, nothing is produced by the team and thus there is nothing available to steal, leaving both with incomes of zero.

The resulting consumption and utility functions are identical to those presented in section 3.1, except that the consumption functions now give us the *expected* consumption, and utility is defined over the latter. We keep the same assumptions as those presented in Assumption 1, and we consider the symmetric subgame perfect pure-strategy equilibrium as before. As in the main model of the paper, the decision to cooperate with members of the other caste will always be unanimous: a productive individual who stays within their own caste and who faces a teammate of the other caste can choose between the expected utility of χz if they do not cooperate and $\left(1-\hat{s}\tau \right)$ if they do, where $\hat{s}$ is the fraction of rent-seekers among individuals in the occupation who are from the other caste. Therefore, the condition for cooperation is $(1-\hat{s}\tau )\geq \chi$; the z cancels out, and thus individuals of all types will make the same decision. We assume that χ>1−0.5τ, which ensures that outcomes with cross-caste rent-seeking will be blocked in stage 1.

The results are straightforward to derive, and identical to those from section 3.3: if occupational mobility is allowed in the first stage, MP/SP is the outcome if τ≤(1+d)∕1.75, and otherwise MP/MR is the result—which will be blocked in the first stage. In the latter case, the {1,1} and {1,2} types both have a choice simply between SP and SR, and the critical value for τ is (1+d+m)∕1.5, leading to the following subgame perfect pure-strategy equilibrium.

*Proposition 5*. The subgame perfect pure-strategy equilibrium to our model with rent-seeking modelled as theft from a teammate takes the following form:

- if τ≤(1+d)∕1.75, the equilibrium will be MP/SP;
- if τ∈((1+d)∕1.75,(1+d+m)∕1.5], the equilibrium will be SP/SP;
- if τ>(1+d+m)∕1.5, the equilibrium will be SR/SR.

This equilibrium is identical to that given by Proposition 1, and additional results available upon request show that the results from the extensions in section 4 also apply to the alternative setting with theft from a teammate, with the exception of a small alteration to the equilibrium in the model featuring an individual choice of education. The essential mechanism is unaffected by the precise interpretation of the process generating immobility: when rent-seeking costs are sufficiently low, some individuals will want to interact with members of other castes in order to engage in rent-seeking, and this generates a lack of trust of members of a different caste, preventing individuals (or the caste leadership in other possible versions of the model) from wanting to engage cooperatively with members of the other caste. The ensuing lack of occupational mobility can then generate even higher levels of rent-seeking in some cases because some individuals have been unable to sort into occupations that suit their talents.
